# Observed magnitude and trends in socioeconomic and geographic area inequalities in obesity prevalence among non-pregnant women in Chad: evidence from three waves of Chad demographic and health surveys

**DOI:** 10.1186/s13690-021-00658-5

**Published:** 2021-07-23

**Authors:** Gebretsadik Shibre, Betregiorgis Zegeye, Gashaw Garedew Woldeamanuiel, Wassie Negash, Gorems Lemma, Mitku Mamo Taderegew

**Affiliations:** 1 Department of Reproductive, Family and Population Health, School of Public Health, Addis Ababa University, Addis Ababa, Ethiopia; 2 HaSET Maternal and Child Health Research Program, Shewarobit Field Office, Shewarobit, Ethiopia; 3 Department of Medicine, College of Medicine and Health Science, Wolketie University, Wolkite, Ethiopia; 4 Department of Public Health, College of Health Science, Debre Berhan University, Debre Berhan, Ethiopia; 5 Chacha Health Center, Angolela Tera Health Office, Chacha, Ethiopia; 6 Department of Biomedical Sciences, College of Medicine and Health Sciences, Wolkite University, P.O. Box 07, Wolkite, Ethiopia

**Keywords:** Geographic, Socioeconomic, Inequality, Trends, Obesity, Women, Chad, DHS

## Abstract

**Background:**

While the prevalence of obesity is increasing worldwide, the growing rates of overweight and obesity in developing countries are disquieting. Obesity is widely recognized as a risk factor for non-communicable diseases (NCDs), including diabetes, cancer and cardiovascular diseases. Available evidence on whether obesity has been more prevalent among higher or lower socioeconomic groups, across regions and urban-rural women’s are inconsistent. This study examined magnitude of and trends in socioeconomic, urban-rural and sub-national region inequalities in obesity prevalence among non-pregnant women in Chad.

**Method:**

Using cross-sectional data from Chad Demographic and Health Surveys (DHSs) conducted in 1996, 2004 and 2014; we used the World Health Organization (WHO) Health Equity Assessment Toolkit (HEAT) to analyze socio-economic, urban-rural and regional inequalities in obesity prevalence among non-pregnant women aged 15–49 years. Inequalities were assessed using four equity stratifiers namely wealth index, educational level, place of residence and subnational region. We presented inequalities using simple and complex as well as relative and absolute summary measures such as Difference (D), Population Attributable Risk (PAR), Population Attributable Fraction (PAF) and Ratio (R).

**Results:**

Though constant pattern overtime, both wealth-driven and place of residence inequality were observed in all three surveys by Difference measure and in the first and last surveys by Ratio measure. Similarly, including the recent survey (D = -2.80, 95% CI:-4.15, − 1.45, *R* = 0.37, 95% CI: 0.23, 0.50) absolute (in 1996 & 2014 survey) and relative (in all three surveys) educational status inequality with constant pattern were observed. Substantial absolute (PAR = -2.2, 95% CI: − 3.21, − 1.34) and relative (PAF = − 91.9, 95% CI: − 129.58, − 54.29) regional inequality was observed with increasing and constant pattern by simple (D) and complex (PAR, PAF) measures.

**Conclusion:**

The study showed socioeconomic and area-based obesity inequalities that disfavored women in higher socioeconomic status and residing in urban areas. Prevention of obesity prevalence should be government and stakeholders’ priority through organizing the evidence, health promotion and prevention interventions for at risk population and general population.

**Supplementary Information:**

The online version contains supplementary material available at 10.1186/s13690-021-00658-5.

## Background

Globally, obesity remains one of the major threats to public health. The emerging burden of chronic non-communicable diseases (NCDs), particularly cardiovascular disease (CVD), diabetes and obesity, threatens the gains in life expectancy made by combating infectious diseases (1, 2). In the African region, where many of these diseases have long been considered “diseases of affluence”, obesity is becoming increasingly prevalent (3, 4). Vulnerable populations are experiencing high double-burdens of infectious and chronic diseases and the emerging burden of obesity in sub-Saharan Africa if not appropriately addressed, in the next decades, will create new challenges to health systems and threaten global economic development of African countries (5, 6).

Recent estimates from the World Health Organization suggest that NCDs kill near 45 million people each year, representing 70% of all deaths globally (7). In Africa, over 115 million people suffer from obesity-related problems and the rates are climbing faster than in just about anywhere else in the world (7). Available evidence suggests that obesity, together with excessive consumption of fat and salt, are risk factors for occurrence of chronic problems such as cancer, chronic kidney disease, diabetes, stroke and heart disease (8). Furthermore, it is well-established that obesity has a detrimental effect on reproductive physiology as it reduces fertility and increase the risk of adverse outcomes for mother and child. Interest for NCDs surveillance had mostly remained the concern of developed countries until the 1990s, when it became evident that the greatest impact of NCDs would be in low- and middle-income countries (LMICs). The 53rd World Health Assembly adopted the “Global strategy for prevention and control of non-communicable diseases”. The resolution positioned surveillance as a key objective of a global strategy, by stressing the need for mapping emerging NCDs epidemics and their determinants with particular reference to poor and disadvantaged populations, in order to provide guidance for policy, legislative and financial measures related to the development of an environment supportive of control (9). The WHO has also adopted a strategy to be implemented by nations worldwide (10) to halt the issue. The strategy put an emphasis on stakeholders’ role in working together to address the health impact (11). As primary prevention, the adoption and implementation of strategies at individual, societal and institutional levels are necessary to effectively prevent obesity and the associated health burdens (10).

While studies have reported associations of obesity with socioeconomic factors among the general population in Chad, the overall prevalence and the associated potential risk factors or the trend has not been assessed (11, 12). There is a dearth of studies examining inequalities in obesity prevalence, and assessing how social structures and processes are critical for equity in achieving healthy weight. Yaya et al. study showed that the prevalence of obesity among women in Chad was 2.3% in 2014 (12). However, such aggregated analyses are not enough to get a clear picture of obesity in the country. Specific evidence from different dimensions of subpopulation within the country in obesity prevalence is important to plan targeted obesity prevention and health promotion intervention and develop policies that can reduce health inequities while improving health for all. There was previous attempt on socio-economic inequality prevalence of obesity (8) that assessed only wealth and education status inequality or it lack evidence on area-based inequality and no information about inequality trends in Chad. This study aimed to address the evidence gap in socioeconomic-related and area-based inequalities in obesity among non-pregnant women in Chad. This paper addressed two research questions: (i) what is the extent of both socioeconomic and area-based inequalities in obesity prevalence among non-pregnant women in Chad and (ii) how were the trends of both socioeconomic and area-based inequalities in obesity prevalence among non-pregnant women in Chad between 1996 and 2014?

## Methods

### Data source

We used cross-sectional data from three rounds of Chad Demographic and Health Surveys (CDHSs) conducted in 1996, 2004 and 2014, which was available in the Health Equity Assessment Toolkit (HEAT) software. HEAT contains World Health Organization (WHO) Health Equity Monitoring (HEM) database (13). The HEM database stores large sets of data conducted from Demographic and Health Surveys (DHS) and Multiple Indicator Cluster Surveys (MICS) in many low-and middle income countries. Including obesity among non-pregnant and non-post-partum women, the database contains more than 30 reproductive, maternal, neonatal and child health indicators, that allows to easily analyze health inequalities (13, 14) All CDHSs are nationally representative surveys that collect information on a wide range of public health related topics such as anthropometric, demographic, socioeconomic, family planning and domestic violence to name a few (15). They were implemented in Chad with the financial and technical assistance by ICF International provisioned through the USAID-funded MEASURE DHS program.

### Selection and measurement of variables

Our interest of outcome variable was prevalence of obesity. The body mass index (BMI) for each woman was calculated as her weight in kilograms divided by the square of her height in meters. Women having a BMI of 30 kg/m2 and above were considered obese, while women with a BMI of less than 30 kg/m2 were classified as not obese (16–18). The outcome variable was categorized in to binary categories as mentioned in the aforementioned sentence and used throughout the analysis. Pregnant and post-partum women as well as women with a BMI of less than 12 or more than 60 were excluded from the analysis (16–18).

Inequality in the prevalence of obesity was measured for four equity stratifiers. Economic status was proxied through a wealth index in the DHS computed using household assets and ownerships following the methodology explained here (19) and was classified in to poorest, poor, middle, rich and richest. The wealth index was computed for each of the three surveys conducted in Chad using principal component analysis (PCA) and was deemed comparable across the survey years. Women’s educational status was classified as no-education, primary education, and secondary education and above, place of residence as urban vs. rural. Sub-national regions included were fifteen, nine and twenty one for 1996, 2004 and 2014 surveys respectively.

### Statistical analysis

The latest version of the WHO’s HEAT software (2019 update) was adopted for the analysis (13). Using the software, the analysis was carried out as follows. First, obesity prevalence was disaggregated by the four equity stratifiers as sex does not apply to our analysis: economic status, educational status, place of residence, and sub-national regions. Finally, we examined inequality in prevalence of obesity using four summary measures; namely Difference (D), Population and Attributable Risk (PAR), Population Attributable Fraction (PAF) and Ratio (R). We selected these summary measures due to their application for all dimension of inequalities. Both simple and complex summary measures were calculated for each equity stratifiers to better understand inequality involved in the occurrence of obesity (13, 14, 20). The Difference and Ratio are simple and un weighted measures of health inequality, whereas the PAR and PAF are complex and weighted measures (13, 14, 20). While simple measures of health inequality are suitable for pairwise comparison of a health indicator of interest, they do not account for the subpopulations in the middle when applied to an equity stratifier with more than two categories, such as wealth index. This issue is avoided by the adoption of complex measures, whereby estimates are based on the sizes of all categories of a particular dimension of inequality (14).

As step-by-step procedures for the calculation of each summary measure included in the health equity database are discussed in detail in the HEAT software technical notes (13) and the WHO handbook on the health inequality monitoring22), only a brief summary is offered here. Summary for education and economic status dimensions of inequality, Difference was calculated as obesity prevalence in the poorest group minus and in the richest group. Summary of the Difference in prevalence between the uneducated group and the group that has acquired at least secondary education was conducted. Similarly, for the place of residence, Difference pertains to that between rural and urban populations, whereas that for the sub-national regions pertains to the Difference between regions with the highest and the lowest obesity prevalence was executed. Except for divide in “ratio” instead of minus, the calculation and references for ratio were similar with difference.

PAR shows the potential for improvement in the national level of obesity prevalence in that could be reduced if all subgroups had the same level of obesity prevalence as a reference subgroup (15, 22). PAR is calculated as the difference between the prevalence of obesity estimate for the reference subgroup y_ref_ and the national average (μ) of prevalence of obesity: PAR = y_ref_ – μ, where y_ref_ refers to the subgroup with the lowest obesity prevalence estimate for binary dimensions (place of residence) and non-ordered dimensions (subnational region and place of residence). For our study, rural for place of residence were the reference. Regarding subnational region, since it was not applicable for 1996, Moyen Chari (in 2004), and Chari Baguirmi (in 2014) regions were the references for calculating PAR since these groups had the lowest prevalence of obesity. For ordered inequality dimensions (economic and education status), y_ref_ refers to the most advantaged subgroups. Hence, richest subgroups for economic status and secondary school and above for educational status were the references.

Similarly, PAF shows the potential for improvement in the national level of obesity prevalence in that could be reduced if all subgroups had the same level of obesity prevalence as a reference subgroup (15). PAF is calculated by dividing the PAR by the national average μ and multiplying the fraction by 100: PAF = [PAR / μ] * 100. Both PAR and PAF takes negative values for adverse health outcome indicators such as obesity, and positive values for favorable indicators such as health service. The larger the absolute value of PAR, the higher the level of inequality. PAR is zero if no further improvement can be achieved, i.e. if all subgroups have reached the same level of obesity prevalence as the reference subgroup (15, 22).

As a measure of statistical significance, 95% Uncertainty Intervals (UI) were computed around point estimates. While interpreting inequality existence, Difference and PAR lower and upper bounds of UI shall not entail zero. R inequality exists if UIs do not involve one. In the case of inequality trend interpretation, UIs of the summary measures for different survey years shall not overlap to conclude a change in inequality over time. We followed the guidelines for Strengthening of Observational studies in Epidemiology (STROBE) during the preparation of this manuscript (21).

To take care of the complex nature of the DHS’s data (14), “svyset” command during analysis and all three design elements such as weight, cluster and strata were taken into consideration (14, 22, 23).

### Ethical consideration

We did the analyses using publicly available data from demographic health surveys. Ethical procedures were the responsibility of the institutions that commissioned, funded, or managed the surveys. All DHS surveys are approved by ICF international as well as an Institutional Review Board (IRB) in respective country to ensure that the protocols are in compliance with the U.S. Department of Health and Human Services regulations for the protection of human subjects.

## Results

### Characteristics of study population

As shown in Table 1, a total of 16, 016 populations were involved in all three DHS rounds. Of them, 77.9 and 19% were rural residents and from wealth quintile 1 subgroups respectively. Regarding educational status, about 65.9 and 20.5% participants were among the no educated and primary school subpopulations respectively. To specifically present the characteristics of sampled population in each round, a total of 3548 and 2940 in 1996 and 2004, and 9528 in 2014 surveys were participated respectively. Approximately 78.5, 80.8 and 75.8% of respondents in 1996, 2004 and 2014 surveys were rural residents respectively. Close to 78.7, 77.4 and 61.1% of respondents in 1996, 2004 and 2014 surveys had no formal education respectively.

**Table 1:** Socioeconomic characteristics of study population (non-pregnant women): Evidence from Chad Demographic and Health Surveys (DHSs)

Variables	Number (%)		
	1996	2004	2014
**Household economic status**			
Quintile 1	700 (20.0)	538 (18.3)	1902 (20.0)
Quintile 2	934 (26.3)	658 (22.4)	1786 (18.7)
Quintile 3	604 (17.0)	586 (19.9)	1818 (19.1)
Quintile 4	663 (18.7)	630 (21.4)	1929 (20.2)
Quintile 5	646 (18.2)	527 (18.0)	2093 (22.0)
**Maternal educational level**			
No education	2791 (78.7)	2276 (77.4)	5825 (61.1)
Primary school	646 (18.2)	528 (18.0)	2212 (23.2)
Secondary school +	110 (3.1)	136 (4.6)	1491 (15.7)
**Place of residence**			
Urban	2785 (78.5)	2376 (80.8)	7220 (75.8)
Rural	763 (21.5)	564 (19.2)	2308 (24.2)

Supplementary file 1 shows study population distribution across subnational region. Out of fifteen regions of the country in 1999, more than half (51.8%) of respondents were from five regions such as Chari Baguirmi (11.5%), Mayokebbi (11.2%), Moyen Chari (11.1%), Ouadda (9.8%) and Ndjamana (8.3%) respectively. In the next survey (2004), out of nine regions more than three-fifth (60.5%) of the participants were from Logone Occidental (22.1%), B.E.T (14.2%), Chari Baguirmi (12.4%), Moyen Chari (11.8%) respectively. And lastly, out of 21 regions participated in 2014 survey, nearly half (46.6%) of respondents were from six regions such as Logone Oriental (9.9%), Ndjamena (9.1%), Mayo Kebbi Est (7.6%), mandoul (6.8%), Logone Occidental (6.7%), and Hadjer-Lamis (6.5%) respectively.

### Prevalence of obesity across subpopulations

The national prevalence of obesity among non-pregnant women was 0.8, 1.6 and 2.4 percentage point in 1996, 2004 and 2014 respectively. Obesity prevalence among non-pregnant women was dissimilar across socio-economic and area-based subgroups in Chad across all three survey periods.

The result shows prevalence of obesity was significantly higher among wealth quintile 5 and 4 respectively as compared to other three wealth quintiles (1, 2 and 3 quintiles). For instance, the prevalence among quintile 5 was 3.6, 5.2 and 7.3 percentage point in 1996, 2004 and 2014 respectively. Whereas, among quintile 1 for example, it was 0, 0.9 and 1.1 percentages point in same survey years respectively.

The pattern of obesity prevalence was different across economic subgroups. It was increasing from 1996 to 2004 and, then continued as constant till 2014 among quintile 1and quintile 2. On the other hand among quintile 5, it was constant from 1996 to 2004, and then it changed in to increasing till 2014. The pattern among quintile 3 and 4 was constant overtime (Table 2).

**Table 2:** Prevalence of obesity among non-pregnant women across socioeconomic and area based subpopulations in Chad from 1996 to 2014

Dimension of Inequality	Subgroup			1996		2004		2014	
				Estimate (95% CI)	Pop^**n**^	Estimate (95% CI)	Pop^**n**^	Estimate (95% CI)	Pop^**n**^
Economic status	Quintile 1 (poorest)			0 (0, 0)	700	0.91 (0.31, 2.62)	538	1.10 (0.64, 1.87)	1902
	Quintile 2			0 (0, 0)	934	0.72 (0.22, 2.29)	658	0.78 (0.43, 1.44)	1786
	Quintile 3			0.10 (0.01, 0.73)	604	0.40 (0.11, 1.41)	586	0.88 (0.49, 1.56)	1818
	Quintile 4			1.02 (0.53, 1.95)	663	1.45 (0.59, 3.51)	630	1.57 (0.89, 2.76)	1929
	Quintile 5 (richest)			3.62 (2.59, 5.02)	646	5.26 (4.03, 6.85)	527	7.39 (6.31, 8.63)	2093
Education	No education			0.39 (0.26, 0.60)	2791	1.37 (0.95, 1.99)	2276	1.65 (1.32, 2.06)	5825
	Primary school			1.91 (1.25, 2.92)	646	2.58 (1.44, 4.57)	528	3.31 (2.29, 4.75)	2212
	Secondary school +			6.54 (3.56, 11.69)	110	2.93 (1.37, 6.17)	136	4.45 (3.32, 5.95)	1491
Place of residence	Rural			0.13 (0.04, 0.43)	2785	0.81 (0.47, 1.39)	2376	0.95 (0.70, 1.28)	7220
	Urban			3.52 (2.70, 4.59)	763	5.26 (4.08, 6.75)	564	7.25 (6.14, 8.55)	2308
Subnational region	01 batha	01 bar azoum	01 batha	0 (0, 0)	161	0.35 (0.07, 1.53)	138	2.92 (1.36, 6.14)	344
	02 b.e.t.	02 b. e. t.	02 borkou/tibesti	2.57 (0.26, 21.02)	23	0.89 (0.31, 2.49)	418	4.52 (2.42, 8.27)	42
	03 biltine	03 centre est	03 chari baguirmi	0 (0, 0)	107	1.03 (0.21, 4.89)	274	0.19 (0.02, 1.37)	379
	04 chari-baguirmi	04 chari baguirmi	04 guera	0.63 (0.14, 2.72)	408	2.18 (1.20, 3.95)	364	0.87 (0.28, 2.67)	524
	05 guara	05 logone occidental	05 hadjer-lamis	0.38 (0.05, 2.84)	161	2.09 (1.11, 3.88)	650	1.32 (0.45, 3.76)	621
	06 kanem	06 mayo kebbi	06 kanem	0.38 (0.05, 2.61)	161	2.31 (0.93, 5.60)	284	0.47 (0.16, 1.36)	365
	07 lac	07 moyen chari	07 lac	0.82 (0.10, 6.05)	155	0.21 (0.03, 1.48)	347	0.48 (0.09, 2.41)	531
	08 logone occidental	08 ouaddai est	08 logone occidental	1.30 (0.33, 4.94)	235	0.49 (0.12, 1.99)	251	4.72 (2.73, 8.04)	641
	09 logone oriental	09 ndjamena	09 logone oriental	0.44 (0.17, 1.13)	279	5.55 (3.78, 8.09)	212	1.55 (0.72, 3.32)	943
	10 mayo-kebbi	NA	10 mandoul	0.62 (0.31, 1.23)	397	NA	NA	1.86 (0.95, 3.60)	649
	11 moyen chari	NA	11 mayo kebbi est	0.62 (0.31, 1.25)	393	NA	NA	0.62 (0.23, 1.61)	722
	12 ouadda	NA	12 mayo kebbi ouest	0.53 (0.27, 1.04)	347	NA	NA	1.40 (0.63, 3.08)	531
	13 salamat	NA	13 moyen chari	0.46 (0.05, 3.74)	132	NA	NA	5.09 (3.63, 7.10)	535
	14 tandjila	NA	14 ouaddai	0 (0, 0)	288	NA	NA	0.42 (0.13, 1.34)	505
	15 ndjamana	NA	15 salamat	4.54 (3.29, 6.23)	293	NA	NA	1.71 (0.67, 4.29)	172
	NA	NA	16 tandjile	NA	NA	NA	NA	2.13 (1.00, 4.48)	574
	NA	NA	17 wadi fira	NA	NA	NA	NA	0.55 (0.10, 2.80)	251
	NA	NA	18 ndjamena	NA	NA	NA	NA	10.12 (8.38, 12.19)	870
	NA	NA	19 barh el gazal	NA	NA	NA	NA	1.54 (0.57, 4.10)	118
	NA	NA	20 ennedi	NA	NA	NA	NA	1.78 (0.72, 4.34)	45
	NA	NA	21 sila	NA	NA	NA	NA	0.31 (0.04, 2.16)	157

Notes: Pop^n^ population, *NA* not available for respective year of survey, *CI* confidence interval

In 1996 survey, obesity prevalence among non-pregnant women was significantly higher among secondary school and above categories followed by primary school subgroups as compared to no educated. Nonetheless, no prevalence difference was observed across education subgroups in 2004 survey. Except in no educated subgroups; prevalence of obesity was significantly lower as compared to the rest two education subgroups, no difference was identified between primary and secondary school and above subgroups in 2014 survey.

With the exception of increasing pattern from 1996 to 2004 among no educated subgroups, the pattern of obesity prevalence among non-pregnant women was constant in other education subgroups and surveys (Table 2). The result from this study also shows presence of significantly higher obesity prevalence among urban residents as compared to their counter parts from 1996 to 2014. The pattern of obesity prevalence among rural residents was increasing from 1996 to 2004 and then, it continued as constant till 2014. However, among urban residents its pattern was constant overtime as presented in Table 2.

Another main finding from the current study is dissimilarity in obesity prevalence across regions within the country in all three surveys. For instance, zero obesity prevalence was observed in 1996 survey in Batha, Biltine and Tandjila regions. In same survey, disproportionately higher prevalence of obesity was observed in B. E. T. next to Ndjamana region. On the other hand, in 2004 survey, the highest and lowest obesity prevalence was observed in Logone Oriental and Lac respectively (Table 2). Due to different regions in all three surveys, figuring out obesity prevalence pattern makes difficult in this study.

### Magnitude and trends of socio-economic and area based inequality

Table 3 shows existence of absolute and relative socio-economic and area-based inequality in obesity prevalence among non-pregnant women in Chad from 1996 to 2014.

**Table 3:** Magnitude and trends in socioeconomic, urban-rural and subnational region inequality in prevalence of obesity among non-pregnant women in Chad: Evidence from Chad Demographic and Health Surveys (1996–2014)

Dimension of inequality	Summary measure	1996	2004	2014
		Estimate (95% CI)	Estimate (95% CI)	Estimate (95% CI)
Economic status	D	−3.6 (−4.80, −2.43)	−4.35 (−6.04, −2.66)	−6.28 (−7.58, −4.99)
	PAF	0 (−85.55, 85.55)	0 (−77.87, 77.87)	0 (−27.89, 27.89)
	PAR	0 (−0.74, 0.74)	0 (−1.29, 1.29)	0 (−0.69, 0.69)
	R	0 (0, 0)	0.17 (−0.01, 0.36)	0.14 (0.06, 0.23)
Education status	D	−6.14 (−10.00, − 2.27)	−1.55 (− 3.80, 0.68)	−2.80 (− 4.15, − 1.45)
	PAF	0 (− 498.41, 498.41)	0 (− 187.39, 187.39)	0 (− 36.19, 36.19)
	PAR	0 (− 4.32, 4.32)	0 (− 3.12, 3.12)	0 (− 0.89, 0.89)
	R	0.06 (0.01, 0.10)	0.46 (0.07, 0.85)	0.37 (0.23, 0.50)
Place of residence	D	−3.38 (−4.33, −2.44)	− 4.44 (−5.82, − 3.06)	− 6.30 (− 7.53, −5.07)
	PAF	0 (−58.53, 58.53)	0 (− 71.94, 71.94)	0 (− 24.44, 24.44)
	PAR	0 (− 0.50, 0.50)	0 (− 1.19, 1.19)	0 (− 0.60, 0.60)
	R	0.03 (−0.00, 0.08)	0.15 (0.06, 0.24)	0.13 (0.08, 0.17)
Subnational region	D	4.54 (3.10, 5.98)	5.34 (3.20, 7.47)	9.92 (7.99, 11.86)
	PAF	−100 (NA, NA)	−87.03 (− 147.24, −26.81)	−91.93 (− 129.58, −54.29)
	PAR	−0.86 (NA, NA)	−1.45 (− 2.45, − 0.44)	−2.27 (− 3.21, − 1.34)
	R	NA	25.70 (− 24.39, 75.79)	50.66 (−47.59, 148.92)

Notes: *D* Difference, *PAR* Population Attributable Risk, *PAF* Population Attributable Fraction, *R* Ratio, *CI* Confidence Interval

Absolute wealth-driven inequality was observed in all three survey years by Difference measure. Furthermore, relative economic inequality was observed in the first (1996) and in the recent survey (2014) by Ratio measure. The pattern of economic inequality by Difference measure was constant overtime. However, no economic inequality was observed by complex measures (PAF, PAR) in all three surveys.

Education based relative inequality in obesity prevalence was observed in all three surveys by Ratio measure whereas absolute educational status inequality in obesity prevalence was observed in 1996 and 2014 surveys only. Similarly, the complex measures (PAR, PAF) didn’t indicate education based inequality in all surveys. Its pattern was constant overtime as described in Table 3 by Ratio measure.

Absolute place of residence inequality was demonstrated from 1996 to 2014 by Difference measure. Likewise, relative urban-rural inequality also observed in 2004 and 2014 by Ratio measure. However, the complex measures didn’t indicate inequality in all three surveys. The pattern of both absolute and relative place of residence inequality in obesity prevalence was constant overtime as presented by Difference and Ratio measures respectively.

Running out the status of regional inequality in 1996 was not applicable. However, according to the next two surveys; 2004 and 2014 surveys, substantial absolute (D, PAR) and relative (PAF) subnational region inequality was identified in both of the surveys. The pattern of absolute inequality was increasing from 2004 to 2014 as described by Difference measure, whereas constant pattern was observed by complex measures (PAF, PAR) (Table 3).

## Discussion

The study sheds light on the extent and time-trend of socio-economic and area-based inequalities in the obesity occurrence among non-pregnant women in Chad using the high-quality WHO health equity monitors database. The overall results showed the presence of marked inequalities in obesity prevalence favoring economically worse-off, uneducated and rural women. Mostly, the UIs of estimates in the adjacent survey years overlap and complicated interpretation of the inequality trends. However, the study confirmed an increasing trend of the inequalities across all equity stratifiers and between the first and the last rounds of surveys.

Based on Difference as a measure of absolute health inequality, the economic status-based inequality assessment indicated that obesity burden is more pronounced among the economically better-off women in each of the three Chad DHS time points. Similar findings were documented in Bangladesh (24–26) and Malawi (27).

The plausible reason behind this might be due to having better purchasing capacities of foods and consuming more diet as well as working in non-laborious occupation type and living sedentary life style among individuals in the higher socioeconomic class (27–31).

. Even if the pattern of absolute economic inequality is constant overtime, if we take and compare the 1996 and 2014 surveys, it has increasing sign. However, no inequality was observed by complex measures (PAR, PAF) in all three surveys. The reason for this might be due to complex measures taking accounts the weights of all subgroups (13, 14) and in this study there was nearly similar obesity prevalence distribution across all wealth quintile in all surveys except quintile 5, which had extremely higher prevalence as compared to quintile 1–4.

The study also revealed education-based disparity in the prevalence of obesity; more educated sub-groups are at higher risk by Difference measure in 1996 and 2014 and in all surveys by Ratio respectively. The occurrence of noticeable inequality in obesity prevalence disproportionately affected the advantaged subpopulations. This conclusion is compatible with the available body of evidence (24, 32, 33). Secondary education or more completed women are more vulnerable to obesity. This could be explained by the fact that educated persons are likely to be recruited to jobs that do not require physical mobility (33). However, by complex measures (PAR and PAF) in all three surveys no education-based inequality exists. In terms of time trend based on the estimates of Ratio, the study indicated that constant pattern was observed in overtime.

Moreover, the study showed that urban-rural disparity in obesity prevalence with higher concentration among women living in urban setting with fairly constant pattern. Studies in Malawi (27), Algeria and Tunisia (34) Botswana (35) in 24 African countries (36) and low and middle income countries (37) reported higher prevalence of obesity among women in urban setting. Evidence shows non frequent physical activities, consumption of sugar-sweetened beverages and high caloric fast food among individual residing in urban areas are main factors for higher burden among these subpopulation (29, 36, 38). Non-availability of natural foods and increased the attraction by western products (may be seen as a status symbol) the urban communities are high likely to consume such sedentary foods and then to be obese than rural (39). This occasionally exacerbated by cultural view in Africa that large body is a sign of prosperity and estimable (40).

Geographical disparities in obesity prevalence were reported in several previous studies (41–43).

Variations of obesity prevalence across regions might be explained partly by differences in resource distribution that facilitate active living (44). Evidence shows regional differences in socioeconomic status across regions might be one of the main reasons for dissimilarities in obesity prevalence (42, 45–47).

Overall, the visible socioeconomic and area-based inequalities in the prevalence of obesity persist over the 18 years. Such evidence is important for the community, policy makers, and concerned stakeholders to address the problem in different ways. It also helps policy makers and stakeholders to plan and design appropriate intervention for at higher risk of obesity population group. Finally, it is important to have prevention strategies and curb the problem through comprehensive approach to address the obesity inequality among all the social groups and this in turn helps to meet and achieve the SDG.

Major implication of this kind of finding is that relying on a single summary measure of inequality might not be enough to better understand an inequality (14). Simple summary measures such as Difference do not tell the whole story of inequality as they are restricted to just two extreme group of a sub-populations and ignore the sub-groups in the middle; this could lead to a conclusion that might be biased especially when there is a population shift in a subpopulation of interest over time (14).

### Strength and limitation of the study

The study has various strengths. First, the inequality analysis in this study was based on the WHO’s high-quality health equity monitor database and this enhanced the quality of the evidence contained in this paper. Also, the study used the 2019 update of the database, so it was possible to capture the current obesity status from information obtained through the latest (2014) round of the CDHS. Second, using of different inequality summary measures in the study might have helped the researchers to exploit the nature of obesity inequality from diverse angles. In other way the imitations were, the study used nationally representative CDHS data, but this finding could not be generalized to areas below the sub-national regions. Also, the WHO equity monitor database does not age-disaggregated the obesity inequality and age should have been used as an equity stratifier to know specific age bracket obesity burden dominates the most. In addition, the study did not decompose the observed obesity inequality to underlying determinants and individual percentage contribution to the inequality of commonly risk factors remains unexplored. In addition, the study did not decompose the observed obesity inequality to underlying determinants and individual percentage contribution to the inequality of commonly risk factors remains unexplored. Moreover, obesity data available in the HEAT software classified as obesity and non-obesity. As a result, the paper lacks detail explanations of obesity in the form of non-overweight, overweight and obesity.

## Conclusions

The study showed both socioeconomic and area-based obesity inequalities disfavored women in the higher socioeconomic status and residing in urban areas. Obesity prevalence inequality was recorded in all the survey years and across all the dimensions of inequality between the 1996 and 2014 CDHS, with constant inequality pattern over time though estimates of the PAR and PAF showed no inequality. Although not applicable to run for 1996 survey and constant subnational region inequality was observed by PAF and PAR, it was increased from 2004 to 2014 by Difference measure. In terms of the sub-national regions, the highest burden of obesity prevalence was identified in Ndjamena in all surveys. Future studies need to go a step forward and estimate the influence of a multitude of determinants on the observed obesity inequality. Prevention of obesity prevalence should be government and stakeholders’ priority through organizing the evidence, health promotion and prevention interventions for at risk population and general population. Stakeholders like health professionals, educators, and media need to support awareness of healthy lifestyle and balanced diets.

## Supplementary Information

**Additional file 1.**

## Data Availability

The datasets generated and/or analyzed during the current study are available in the WHO’s HEAT version 3.1 [https://www.who.int/gho/health_equity/assessment_toolkit/en/.].

## Abbreviations

CDHS: Burundi Chad Demographic and health Survey
CI: Confidence Interval
D: Difference
HEAT: Health Equity Assessment Toolkit
PAF: Population Attributable Fraction
PAR: Population Attributable Risk
PPS: Probability Proportional to Size
R: Ratio
SDG: Sustainable Development Goal
UI: Uncertainty Interval
WHO: World Health Organization
