# Supplementary material for: Observed magnitude and trends in socioeconomic and geographic area inequalities in obesity prevalence among non-pregnant women in Chad: evidence from three waves of Chad demographic and health surveys
Source: Arch Public Health. 2021 Jul 23;79:133. doi: 10.1186/s13690-021-00658-5 (PMC8299664; doi:10.1186/s13690-021-00658-5)
Supplement: Supplementary file 1 — Additional file 1. [file 13690_2021_658_MOESM1_ESM.docx]

**Supplementary file 1**

| **Subnational region** | | | **Year** | | |
| --- | --- | --- | --- | --- | --- |
| **1996** | **2004** | **2014** | **1996** | **2004** | **2014** |
| 01 Batha | 01 Bar Azoum | 01 Batha | 161 | 138 | 344 |
| 02 B.E.T. | 02 B. E. T. | 02 Borkou/Tibesti | 23 | 418 | 42 |
| 03 Biltine | 03 Centre Est | 03 Chari Baguirmi | 107 | 274 | 379 |
| 04 Chari-Baguirmi | 04 Chari Baguirmi | 04 Guera | 408 | 364 | 524 |
| 05 Guara | 05 Logone Occidental | 05 Hadjer-Lamis | 161 | 650 | 621 |
| 06 Kanem | 06 Mayo Kebbi | 06 Kanem | 161 | 284 | 365 |
| 07 Lac | 07 Moyen Chari | 07 Lac | 155 | 347 | 531 |
| 08 Logone Occidental | 08 Ouaddai Est | 08 Logone Occidental | 235 | 251 | 641 |
| 09 Logone Oriental | 09 Ndjamena | 09 Logone Oriental | 279 | 212 | 943 |
| 10 Mayo-Kebbi | NA | 10 Mandoul | 397 | NA | 649 |
| 11 Moyen Chari | NA | 11 Mayo Kebbi Est | 393 | NA | 722 |
| 12 Ouadda | NA | 12 Mayo Kebbi Ouest | 347 | NA | 531 |
| 13 Salamat | NA | 13 Moyen Chari | 132 | NA | 535 |
| 14 Tandjila | NA | 14 Ouaddai | 288 | NA | 505 |
| 15 Ndjamana | NA | 15 Salamat | 293 | NA | 172 |
| NA | NA | 16 Tandjile | NA | NA | 574 |
| NA | NA | 17 Wadi Fira | NA | NA | 251 |
| NA | NA | 18 Ndjamena | NA | NA | 870 |
| NA | NA | 19 Barh El Gazal | NA | NA | 118 |
| NA | NA | 20 Ennedi | NA | NA | 45 |
| NA | NA | 21 Sila | NA | NA | 157 |

**Title:** Study population’s distribution across subnational regions for prevalence of obesity among non-pregnant women: Evidence from Chad Demographic and Health Surveys (1996-2014)
